# TaqMan-quantitative PCR assays applied in *Neospora caninum* knock-outs generated through CRISPR-Cas9 allow to determine the copy numbers of integrated dihydrofolate reductase-thymidylate synthase *drug selectable markers*

**DOI:** 10.3389/fcimb.2024.1419209

**Published:** 2024-06-21

**Authors:** Laura Rico-San Román, Kai Pascal Alexander Hänggeli, Andrew Hemphill, Pilar Horcajo, Esther Collantes-Fernández, Luis Miguel Ortega-Mora, Ghalia Boubaker

**Affiliations:** ^1^ SALUVET, Animal Health Department, Complutense University of Madrid, Madrid, Spain; ^2^ Institute for Parasitology, Vetsuisse Faculty, University of Bern, Bern, Switzerland

**Keywords:** duplex TaqMan-qPCR, single TaqMan-qPCR, DHFR-TS, mdhfr-ts, CRISPR-Cas9, off target effects, *Neospora caninum*, *Toxoplasma gondii*

## Abstract

As for many other organisms, CRISPR-Cas9 mediated genetic modification has gained increasing importance for the identification of vaccine candidates and drug targets in *Neospora caninum*, an apicomplexan parasite causing abortion in cattle and neuromuscular disease in dogs. A widely used approach for generating knock-out (KO) strains devoid of virulence factors is the integration of a drug selectable marker such as mutated dihydrofolate reductase-thymidylate synthase (*mdhfr-ts*) into the target gene, thus preventing the synthesis of respective protein and mediating resistance to pyrimethamine. However, CRISPR-Cas9 mutagenesis is not free of off-target effects, which can lead to integration of multiple *mdhfr-ts* copies into other sites of the genome. To determine the number of integrated *mdhfr-ts* in *N. caninum*, a duplex quantitative TaqMan PCR was developed. For this purpose, primers were designed that amplifies a 106 bp fragment from wild-type (WT) parasites corresponding to the single copy *wtdhfrs-ts* gene, as well as the mutated *mdhfrs-ts* present in KO parasites that confers resistance and were used simultaneously with primers amplifying the diagnostic *NC5* gene. Thus, the *dhfr-ts* to *NC5* ratio should be approximately 1 in WT parasites, while in KO parasites with a single integrated *mdhrf-ts* gene this ratio is doubled, and in case of multiple integration events even higher. This approach was applied to the *Neospora* KO strains Nc*ΔGRA7* and Nc*ΔROP40*. For Nc*ΔGRA7*, the number of tachyzoites determined by *dhfr-ts* quantification was twice the number of tachyzoites determined by *NC5* quantification, thus indicating that only one *mdhfr-ts* copy was integrated. The results obtained with the Nc*ΔROP40* strain, however, showed that the number of *dhfr-ts* copies per genome was substantially higher, indicating that at least three copies of the selectable *mdhfr-ts* marker were integrated into the genomic DNA during gene editing by CRISPR-Cas9. This duplex TaqMan-qPCR provides a reliable and easy-to-use tool for assessing CRISPR-Cas9 mediated mutagenesis in WT *N. caninum* strains.

## 1 Introduction

*Neospora caninum* is an obligate intracellular apicomplexan cyst-forming parasite and is closely related to *Toxoplasma gondii* (Dubey and Schares, 2011; Dubey et al., 2017). It is a significant veterinary pathogen and the etiological agent of bovine neosporosis, which is one of the main causes of cattle abortion worldwide (Lindsay and Dubey, 2020; Hecker et al., 2023), resulting in significant economic losses (Reichel et al., 2013). Currently, there are no vaccines or drugs available to treat the disease, and the knowledge of the molecular mechanisms involved in the virulence of parasite is limited.

In this respect, functional genomics can aid in the reliable characterization of potential parasite virulence factors, as well as in the identification of vaccine candidates and drug targets. Validation of such factors is usually carried out by the generation of knockouts (KOs) that are deficient in their expression. In this context, the development of genetic manipulation methods has been crucial in advancing our understanding of the biology of apicomplexan parasites (Suarez et al., 2017).

In 2018, the CRISPR-Cas9 technology was successfully applied to *N. caninum*, using the same constructs previously developed for *T. gondii* (Arranz-Solís et al., 2018). Subsequently, an increased number of mutant parasites deficient in certain genes have been developed by disrupting the target gene sequence by incorporation of a selection marker. The most frequently used selection marker is the modified dihydrofolate reductase-thymidylate synthase (*mdhfr-ts*) (Nishikawa et al., 2018; Zhao et al., 2020; Dong et al., 2021; Wang et al., 2021; Yang et al., 2021), which provides resistance to pyrimethamine, allowing the selection of mutant parasites that grow in the presence of pyrimethamine.

Generation of KO mutants is followed by verification of target gene editing (Zhang et al., 2015). However, it is also important to consider off-target effects (OTEs), such as multiple insertions of the *mdhfr-ts* marker and OTEs are often overlooked. While the CRISPR-Cas9 system is highly effective, achieving absolute specificity remains a challenge. Despite efforts to enhance precision, excision may occur at various locations within the genome due to the presence of PAM sequences (Di Cristina and Carruthers, 2018), DNA/RNA bulges (Lin et al., 2014), electroporation conditions (Alkan et al., 2018), as well as through unspecific targeting by the gRNAs themselves (Jinek et al., 2012).

Recently, in order to identify OTEs resulting from multiple insertion of the selection marker in *T. gondii* KO strains generated by CRISPR-Cas9, a duplex-TaqMan qPCR-based approach has been developed (Hänggeli et al., 2022). *N. caninum* shares a high degree of homology and synteny with *T. gondii* (Reid et al., 2012). Thus, the purpose of this work was to develop and validate a duplex TaqMan-qPCR for *N. caninum* to assess *mdhfr-ts* copy numbers inserted into the genome of CRISPR-Cas9-generated *N. caninum* mutants.

## 2 Materials and methods

### 2.1 Parasite culture and generation of KO strains

The parasite strains used in this study were the *N. caninum* Nc-Spain7 isolate (wild-type strain, WT) and the KO strains Nc*ΔGRA7 and* Nc*ΔROP40*, which are defective in the Nc*GRA7* and Nc*ROP40* genes, respectively. These KO parasites were previously generated using CRISPR-Cas9 technology, by deleting the target gene with two gRNAs directed to the 5’ and 3’ end of the gene and replacing it with the pLoxP-mCherry-DHFR plasmid (Addgene Plasmid #70147; P-*mDHFR*) (Arranz-Solís et al., 2018; Rico-San Román et al., 2022). The P-*mDHFR* plasmid contains the modified *Toxoplasma DHFR-TS* pyrimethamine-resistant allele marker (*mdhfr-ts*), which allows the selection of mutant parasites by adding pyrimethamine in cell culture medium (10 µM). Tachyzoites were routinely *in vitro* maintained by continuous passage in a monolayer culture of the MARC-145 cell line, as previously described (Regidor-Cerrillo et al., 2011; Rico-San Román et al., 2023).

Finally, the mutant parasites were analyzed by PCR to confirm deletion of the targeted gene and its replacement by the *mdhfr-ts* (Arranz-Solís et al., 2018; Rico-San Román et al., 2022). Additionally, Western blotting (WB) and Immunofluorescence assay (IFA) were used to confirm the lack of protein expression in KO tachyzoites compared to WT parasites, as described (Rico-San Román et al., 2022).

### 2.2 Design of primers and probe

To determine the copy numbers of the inserted *mdhfr-ts* selectable marker in the genome of KO clones, we designed a TaqMan-qPCR taking advantage of the fact that WT tachyzoites have a single copy of *dhfr-ts* in their genome (*wtdhfr-ts).* The copy number of the *dhfr-ts* fragment in a given DNA quantity of KO parasites with a single site-specific integration should be twice the number of *dhfr-ts* copies recorded in the same DNA quantity from WT parasites.

The *dhfr-ts* DNA sequences coding for *T. gondii* RH and *N. caninum* were retrieved from ToxoDB database (TGRH88_062920 and NCLIV_065390). Sequences were aligned, and different primers were designed in exon 7: a forward primer (5’-ATCTGGGACAAGAATGTGAC-3’), a reverse primer (5’-CGAAGTGTCTCCACTGGA-3’) and a TaqMan probe (Cy5-CGAGAGGTCGGAGACATCGGC-BHQ). Nucleotide sequences for primers and probe were designed in 100% conserved between *T. gondii dhfr-ts*, *N. canium dhfr-ts*, plasmid P972 (Hänggeli et al., 2022) and P-*mDHFR*. Plasmid P972 was previously used by Hänggeli et al. for amplification of the selectable marker *mdhfr-ts* and generation of *sag1*-defective *T. gondii* KO mutants (*T. gondii* RH *Δsag1*).

### 2.3 Validation of the designed primers and probe by TaqMan q-PCR

Specific *dhfr-ts* forward and reverse primers were designed to amplify a 106 bp fragment and were tested by conventional PCR and agarose gel using DNA from WT*-T. gondii* RH, *T. gondii* RH *Δsag1* C18, WT-*N. caninum*, plasmid P972 and plasmid P-*mDHFR*. The PCR was performed in 25 µl final volume containing 12.5 µl of GoTaq^®^ G2 Master Mix (Promega, Madison, WI, USA), 0.5 µl of each primer (forward and reverse, 50 µM), 10.5 µl of distilled water and 1 µl of template DNA. Conditions were as follows: an initial denaturation step at 98°C for 3 min, followed by 25 cycles of denaturation at 95°C for 5 min, annealing at 58°C for 30 sec, and elongation at 72°C for 20 sec. The final cycle was followed by an extension step at 72°C for 2 min.

Subsequently, *dhfr-ts* primers were validated by qPCR. DNA controls of WT-*T. gondii* RH and *T. gondii* RH *Δsag1* C18 were used (Hänggeli et al., 2022). The C18 clone was selected as a certified control for single insertion of the complete *mdhfr-ts* selectable marker. PCR amplification was performed using a Bio-Rad CFX 96 qPCR instrument (Bio-Rad, Hercules, CA, USA) in a total reaction mixture of 10 μL containing 1x SensiFast master mix (Bioline, Meridian Bioscience, Cincinnati, OH, USA), 0.5 μM of reverse and forward primers, 0.1 μM of DHFRQ-P probe, 0.3 mM dUTP, one unit of heat-labile Uracil DNA Glycosylase (UDG) (Longo et al., 1990) and 5 ng DNA template. The following thermal profile was applied: an initial incubation of 10 min at 42°C, followed by a denaturation step of 5 min at 95°C and 50 cycles of two-step amplification (10 sec at 95°C and 20 sec at 62°C). Samples were tested in triplicates and a negative control with double-distilled water was included for each experiment. For quantification, a standard curve based on the use of a 10-fold serial dilution of DNA from WT-*T. gondii* RH was used, with tachyzoite numbers ranging from 10^5^ to 10^1^ per 3 μL.

### 2.4 Duplex TaqMan-qPCR targeting *dhfr-ts* and *NC5*

Once the TaqMan-qPCR was validated, the number of tachyzoites corresponding to a given DNA quantity and the copy number of the *dhfr-ts* DNA fragment in the same given quantity were assessed simultaneously. Quantification of tachyzoites was achieved by preparation of a *N. caninum* standard curve, using 10-fold serial dilutions with parasite concentrations ranging from 8.5 x 10^4^ to 8.5 ng/μL and amplification of a 338 bp region of the *N. caninum NC5* gene (Collantes-Fernández et al., 2002). Amplifications were carried-out in total volume of 10 μL containing 1 x SensiFast master mix (Bioline, Meridian Bioscience, Cincinnati, OH, USA), 0.5 μM of each primer set (i: NC5-F 5’-CCCAGTGCGTCCAATCCTGTAAC-3’ and NC5-R 5’-CTCGCCAGTCAACCTACGTCTTCT-3’; ii: dhfr-F/R), 0.1 μM of each probe (DHFRQ-P Cy5 and NCQ-PFAM-TGGTAGCGGTGAGAGGTGGGATACGTG-BHQ), 0.3 mM dUTP, and one unit of UDG. From each sample, 4.25 ng of DNA were used in the reaction mix. All reactions were run in triplicates and amplifications were carried-out under the same thermal profile used for the single TaqMan-qPCR. The cycle threshold values (CT) were plotted as mean of triplicates against the standard curve values to determine the number of tachyzoites. Parasite concentrations were determined after the calculation of the linear regression equation (y = ax + b), where y = CT; a = curve slope (slope); x = parasite number; and b = where the curve intersects y-axis (y intercept). Then, the whole experiment test with duplex TaqMan qPCR was repeated with 8.5 ng of DNA for validating results.

## 3 Results

### 3.1 Validation of designed primers for the duplex TaqMan q-PCR

**Figure 1:**
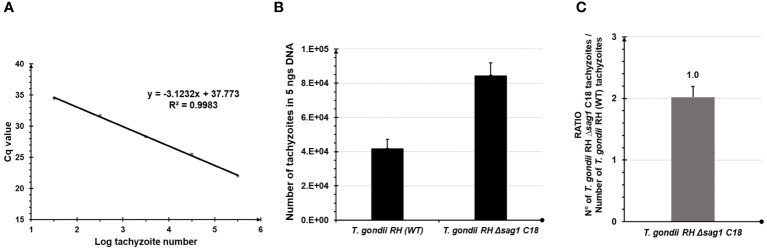
Validation of designed primers for determining the copy number of integrated *mdhfr-ts* marker using *Toxoplasma gondii* samples and a single TaqMan-qPCR. **(A)** WT-*T.gondii* RH standard curve. **(B)** Number of tachyzoites in 5 ng DNA determined using the specific *dhfr-ts* forward and reverse primers designed for *Neospora caninum* knock-out samples and the WT-*T.gondii* RH standard curve. Error bars indicate standard deviations of triplicates for each sample. **(C)** Number of inserted *mdhfr-ts* in *T.gondii* RH *Δsag1* C18 calculated using the ratio of the number of *T.gondii* RH *Δsag1* C18 tachyzoites and the number of WT-*T.gondii* RH tachyzoites determined by *dhfr-ts* amplification. Error bars indicate standard deviations of triplicates for each sample.

Assessment of the specificity of primers to amplify a 106 bp fragment of *wtdhfr-ts* in *N. caninum* and *T. gondii* RH wild type strains, and *mdhfr-ts* in plasmids P972 and P-*mDHFR*, resulted in the amplification of one band with expected size in all samples. Subsequently, the primers were tested to quantify numbers of *dhfr-ts* copies in the genome of WT-*T. gondii* RH parasites and the *T. gondii* RH *Δsag1* C18 mutant parasites. As show in **Figure 1**, the number of tachyzoites estimated in 5 ng of DNA extracted from *T. gondii* RH *Δsag1* C18 was twice that estimated in 5 ng of DNA extracted from *T. gondii* RH. This indicates that *T. gondii RH Δsag1* C18 tachyzoites harbor two copies of the *dhfr-ts* gene -one is the *wtdhfr-ts*, and the other is the integrated *mdhfr-ts* conferring pyrimethamine resistance-, thus revealing a single insertion of the m*dhfr-ts* selectable marker in their genome. These results confirm previous findings and validate the designed primers.

Data and detailed calculations are shown in **Supplementary Table 1**.

### 3.2 Duplex TaqMan qPCR

The two *N. caninum* KO strains (Nc*ΔGRA7* and Nc*ΔROP40)* were tested in the duplex TaqMan qPCR by applying the same principle described by Hänggeli et al., 2022. For *N. caninum* WT parasites, the number of tachyzoites determined by the amplification of *dhfr* is equal to the number of tachyzoites determined by amplification of *NC5*, resulting in a ratio of 1. In case of a KO with single insertion of the *mdhfr-ts*, the number of tachyzoites given by the *dhfr* qPCR is the double of the number of tachyzoites determined by *NC5*, resulting in a ratio of 2. In case of multiple insertion, the ratio will be > 2.

**Figure 2:**
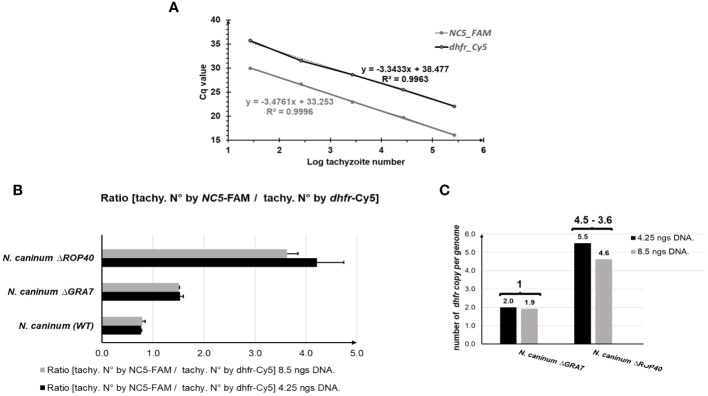
TaqMan-qPCR for determining the copy number of integrated *mdhfr-ts* selectable marker. **(A)** *Neospora caninum* (WT) standard curve amplified with *dhfr* (black line) and *NC5* (grey line) primers. **(B)** Numer of *dhfr* copy per genome given by the ratio of the number of tachyzoites determined by using the *NC5* primer and the number of tachyzoites determined by *dhfr* amplification, using 2 different DNA concentrations: 8.5 ng DNA (grey bar) and 4.25 ng DNA (black bar). Error bars indicate standard deviations of triplicates for each sample. **(C)** Number of inserted *mdhfr-ts* marker in the *N. caninum* mutants knock-out (KO). Bars indicate the ratios calculated in **(B)** and numbers above brackets indicate the calculated number of *mdhfr-ts* copies inserted in each KO. This number is defined by substracting the *dhfr-ts* copy number of the WT, 1, from the ratio calculated for each KO. The result of 1 in Nc*ΔGRA7* indicates a single integration event of *mdhfr-ts*. However, the result of 4.5–3.6 in Nc*ΔROP40* indicates multiple integration events of *mdhfr-ts*.

As shown in **Figure 2**, the standard curves performed for the two target genes, *NC5* and *dhfr*, had similar slopes and efficiencies (*dhfr*-slope = -3.34 and R^2^ = 0.9963 and NC5-slope = -3.47 and R^2^ = 0.9996).

Also, the mean number of *N. caninum* WT tachyzoites determined by *NC5* amplification was similar to the mean number of tachyzoites determined by *dhfr-ts* amplification for the tested DNA quantities (4.25 ng and 8.5 ng), resulting thus in a ratio of 0.8 (**Figure 2B**). For the KO parasites Nc*ΔGRA7*, the number of tachyzoites determined by *dhfr-ts* quantification was twice the number of tachyzoites determined by *NC5* quantification, thus resulting in a ratio of 1.5 for both quantities (**Figure 2B**). This ratio is the double of WT ratio (0.8), indicating a single insertion of the *mdhfr-ts* selectable marker (**Figure 2C**). However, in the Nc*ΔROP40* strain, for both DNA quantities analyzed, the number of *dhfr-ts* copies per genome was higher than 2 (5.5 and 4.6), indicating that at least three copies of the selectable *mdhfr-ts* marker were integrated into the genomic DNA during gene editing by CRISPR-Cas9 (**Figures 2B, C**).

Detailed results and calculation for the duplex TaqMan qPCR are included in **Supplementary Table 2**.

## 4 Discussion

It is imperative to examine *N. caninum* KO mutants for OTEs due to the insertion of multiple *dhfr-ts* copies, especially when employing a gene replacement strategy with two gRNAs. In the CRISPR-Cas9 system, gRNA(s) sometimes bind other than target loci (Pacesa et al., 2022). Off-target sites represent a significant challenge, particularly when the targeted gene belongs to a gene family such as ROP40, a member of rhoptry family protein (Camejo et al., 2014). Therefore, tools are needed to identify whether off-target insertion of the selection marker occurs in the genome.

Previously, Hänggeli et al. designed a single and a duplex TaqMan-qPCR to determine the copy number of the integrated *mdhfr-ts* selection marker during site-specific mutagenesis in *T. gondii* by CRISPR-Cas9 (Hänggeli et al., 2022). However, this qPCR could not be directly used in *N. caninum* for the following reasons: i) when aligning *T. gondii wt dhfr-ts* and *N. caninum wt dhfr*-ts sequences, it became evident that several single nucleotide polymorphisms occurred in the forward/reverse primers and in the probe, which would affect the accuracy of the qPCR; ii) it is not possible to use the *T. gondii* diagnostic PCR based on the 529 bp repeat element for the quantification of *N. caninum* in the duplex qPCR. Thus, the *Neospora*-specific diagnostic qPCR based on *NC5* had to be used, and conditions had to be adapted.

Although the single qPCR can be applied on its own, it is recommended to perform multiplexing with the *NC5*-qPCR. The determination of *dhfr-ts* copy numbers in the KOs samples using the single *dhfr*-qPCR is completely dependent on the WT DNA sample. Therefore, it is important to extract DNA from the same number of parasites for all samples. However, there is still a risk that the amount of DNA from host cells may not be consistent in all DNA preparation, even if the tachyzoites were column purified before counting and DNA purification. In the duplex qPCR, a ratio is calculated for each sample independently of the WT, as explained in Hänggeli et al., 2022. For this reason, the single PCR was used to validate the primers and probe using certified *T. gondii* WT and KO DNA controls for single insertion.

In this work, the duplex qPCR for detecting the *mdhfr-ts* selection marker was successfully adapted to be used in *N. caninum*. The results showed that the Nc*ΔGRA7* strain harbors a single *mdhfr-ts* insertion in the genome, located at the site of the deleted gene of interest, as found in a previous study (Rico-San Román et al., 2022). However, multiple insertions of the *mdhfr-ts* cassette were detected in the Nc*ΔROP40* strain. Upon generation of the Nc*ΔROP40* strain, the fragment corresponding to the *ROP40* gene was found to be correctly disrupted by a single *mdhfr-ts* insertion (Rico-San Román et al., 2022). However, whole genome sequencing was not performed, thus introduction of other *mdhfr-ts* copies in the genome was not detected. Nevertheless, complementation of Nc*ΔROP40* resulted in restoration of virulence (Rico-San Román et al., 2022), thus the additional *mdhfr-ts* copies inserted elsewhere in the genome did not produce phenotypic changes.

Multiple insertions of the selection cassette out of the targeted region are not problematic for the KO phenotype if the multiple integration occurs in non-coding DNA regions, especially if the complemented KO allows recovery of the WT phenotype. However, multiple insertion of the selection cassette, particularly *mdhfr-ts*, could be a concern if it leads to significantly high levels of the active mDHFR-TS enzyme, which plays a crucial role in pyrimidine synthesis (Rosowsky et al., 2003; Hopper et al., 2019). This issue is still largely unknown, in particular the impact of multiple insertion of *mdhfr-ts* on parasite growth and gene expression pattern. This aspect may contribute to the observed differences in the behavior of *GRA7*-deficient parasites generated by different groups using CRISPR-Cas9 and the selection marker *mdhfr-ts* (Nishikawa et al., 2018; Wang et al., 2021; Abdou et al., 2022; Rico-San Román et al., 2022). It is therefore important to develop tools that allow to determine the number of *mdhfr-ts* copies inserted in mutant parasites, such as the duplex qPCR presented herein. Its use in the generation and selection protocols of mutants and the identification of those with a single insertion of the selection marker, will help to achieve greater homogeneity and reliability of the results between groups.

## Data availability statement

The original contributions presented in the study are included in the article/**Supplementary Material**. Further inquiries can be directed to the corresponding author/s.

## Author contributions

LR: Formal analysis, Investigation, Visualization, Writing – original draft. KH: Methodology, Writing – review & editing. AH: Resources, Writing – review & editing. PH: Funding acquisition, Writing – review & editing. EC-F: Funding acquisition, Writing – review & editing. L-MO-O: Funding acquisition, Resources, Writing – review & editing. GB: Conceptualization, Formal analysis, Methodology, Supervision, Visualization, Writing – original draft.
