# Supplementary material for: TaqMan-quantitative PCR assays applied in Neospora caninum knock-outs generated through CRISPR-Cas9 allow to determine the copy numbers of integrated dihydrofolate reductase-thymidylate synthase drug selectable markers
Source: Front Cell Infect Microbiol. 2024 Jun 21;14:1419209. doi: 10.3389/fcimb.2024.1419209 (PMC11224286; doi:10.3389/fcimb.2024.1419209)
Supplement: Supplementary file 1 [file Table_1.docx]

Supplementary Material

Supplementary Table 1. Data and calculations obtained from single TaqMan-qPCR primer validation.

| Single TaqMan-qPCR _*dhfr* | | | | | | | | | |
| --- | --- | --- | --- | --- | --- | --- | --- | --- | --- |
| slope | -3.123 | | | | | | | | |
| y-intercept | 37.77 | | | | | | | | |
| **Label** | **Cq** | **Log tachy. N°** | **tachy. N°** | **Mean of tachy. N°** | **St dev of tachy. N°** | **Ratio**  **[tachy. N° in KO / mean of tachy. N° in WT]** | **Mean of the ratio ± SD** | **N° of *dhfr* copies/genome** | **N° of inserted *dhfr*** |
| *T. gondii* RH (WT) | 23.22 | 4.66 | 4.56E+04 | 4.19E+04 | 5.28E+03 |  |  | 1 |  |
| *T. gondii* RH (WT) | 23.22 | 4.66 | 4.56E+04 |  |  |  |  |  |  |
| *T. gondii* RH (WT) | 23.60 | 4.54 | 3.44E+04 |  |  |  |  |  |  |
| *T. gondii* RH *Δsag1* C18 | 22.24 | 4.97 | 9.42E+04 | 8.46E+04 | 7.27E+03 | 2.25 | 2.02 ± 0.17 | 2.02 | 1.02 |
| *T. gondii* RH *Δsag1* C18 | 22.41 | 4.92 | 8.30E+04 |  |  | 1.98 |  |  |  |
| *T. gondii* RH *Δsag1* C18 | 22.52 | 4.88 | 7.66E+04 |  |  | 1.83 |  |  |  |

Supplementary Table 2. Data and calculations obtained from duplex TaqMan-qPCR using two different amounts of DNA.

| **Duplex TaqMan-qPCR** | | | | | | | | | | | | | | | |
| --- | --- | --- | --- | --- | --- | --- | --- | --- | --- | --- | --- | --- | --- | --- | --- |
| **DHFR-PRIMERS/PROBE** | | | | | | **NC5-PRIMERS/PROBE** | | | | | ***dhfr*-CY5/nc5-FAM** | | | | |
| slpoe | -3.343 | | | | | slpoe | -3.476 | | | |  |  |  |  |  |
| y-intercept | 38.47 | | | | | y-intercept | 37.25 | | | |  |  |  |  |  |
| Label | Cq (CY5) | Log tachy. N° (CY5) | tachy. N° (CY5) | Mean tachy. N° (CY5) | SD | Cq (FAM) | Log tachy. N° (FAM) | tachy. N° (FAM) | Mean tachy. N° (FAM) | SD | Tachy. N°  [CY5)/FAM] | MEAN Tachy. N° [CY5)/FAM] | SD | N° of *dhfr* per genome | N° of integrated *DHFR* |
| **DNA_imput 4.25 ngs** | | | | | | | | | | |  |  |  |  |  |
| *N. caninum* (WT) | 22.5 | 4.73 | 5.41E+04 | 5.37E+04 | 6.58E+02 | 16.4 | 4.8 | 7.05E+04 | 7.02E+04 | 7.00E+02 | 0.77 | 0.8 | 0.0 |  |  |
| *N. caninum* (WT) | 22.6 | 4.72 | 5.27E+04 |  |  | 16.4 | 4.9 | 7.08E+04 |  |  | 0.74 |  |  |  |  |
| *N. caninum* (WT) | 22.5 | 4.73 | 5.41E+04 |  |  | 16.4 | 4.8 | 6.92E+04 |  |  | 0.78 |  |  |  |  |
| *N. caninum ∆GRA7* | 23.3 | 4.53 | 3.35E+04 | 3.52E+04 | 2.13E+03 | 18.1 | 4.4 | 2.29E+04 | 2.30E+04 | 1.77E+03 | 1.47 | 1.5 | 0.1 | 2.0 | 1.0 |
| *N. caninum ∆GRA7* | 23.2 | 4.53 | 3.39E+04 |  |  | 18.2 | 4.3 | 2.10E+04 |  |  | 1.62 |  |  |  |  |
| *N. caninum ∆GRA7* | 23.1 | 4.58 | 3.82E+04 |  |  | 17.9 | 4.4 | 2.53E+04 |  |  | 1.51 |  |  |  |  |
| *N. caninum ∆ROP40* | 21.0 | 5.17 | 1.47E+05 | 1.69E+05 | 2.18E+04 | 17.5 | 4.5 | 3.30E+04 | 4.01E+04 | 5.30E+03 | 4.45 | 4.3 | 0.5 | 5.6 | 4.6 |
| *N. caninum ∆ROP40* | 20.9 | 5.21 | 1.61E+05 |  |  | 17.0 | 4.7 | 4.58E+04 |  |  | 3.53 |  |  |  |  |
| *N. caninum ∆ROP40* | 20.6 | 5.30 | 1.99E+05 |  |  | 17.2 | 4.6 | 4.15E+04 |  |  | 4.79 |  |  |  |  |
| **DNA_imput 8.5 ngs** | | | | | | | | | | | | | | | |
| *N. caninum* (WT) | 21.2 | 5.12 | 1.33E+05 | 1.27E+05 | 3.99E+03 | 15.0 | 5.2 | 1.76E+05 | 1.63E+05 | 1.53E+04 | 0.8 | 0.8 | 0.1 |  |  |
| *N. caninum* (WT) | 21.3 | 5.10 | 1.27E+05 |  |  | 15.1 | 5.2 | 1.71E+05 |  |  | 0.7 |  |  |  |  |
| *N. caninum* (WT) | 21.3 | 5.09 | 1.23E+05 |  |  | 15.3 | 5.2 | 1.42E+05 |  |  | 0.9 |  |  |  |  |
| *N. caninum ∆GRA7* | 21.4 | 5.06 | 1.14E+05 | 1.14E+05 | 4.17E+02 | 16.3 | 4.9 | 7.49E+04 | 7.55E+04 | 5.23E+02 | 1.5 | 1.5 | 0.0 | 1.9 | 0.9 |
| *N. caninum ∆GRA7* | 21.4 | 5.05 | 1.13E+05 |  |  | 16.3 | 4.9 | 7.61E+04 |  |  | 1.5 |  |  |  |  |
| *N. caninum ∆GRA7* | 21.4 | 5.06 | 1.14E+05 |  |  | 16.3 | 4.9 | 7.56E+04 |  |  | 1.5 |  |  |  |  |
| *N. caninum ∆ROP40* | 20.0 | 5.47 | 2.94E+05 | 2.80E+05 | 2.56E+04 | 16.2 | 4.9 | 8.14E+04 | 7.69E+04 | 3.92E+03 | 3.6 | 3.6 | 0.2 | 4.6 | 3.6 |
| *N. caninum ∆ROP40* | 19.9 | 5.48 | 3.02E+05 |  |  | 16.3 | 4.9 | 7.74E+04 |  |  | 3.9 |  |  |  |  |
| *N. caninum ∆ROP40* | 20.3 | 5.39 | 2.44E+05 |  |  | 16.4 | 4.9 | 7.18E+04 |  |  | 3.4 |  |  |  |  |
